# Tumor Mutational Burden and Genomic Alterations in Chinese Small Cell Lung Cancer Measured by Whole-Exome Sequencing

**DOI:** 10.1155/2019/6096350

**Published:** 2019-11-06

**Authors:** Shan Su, Jian-Jun Zou, Yun-Yun Zeng, Wen-Chang Cen, Wei Zhou, Yan Liu, Duo-Hua Su, Xian-Lan Zhang, Hui-Yi Huang, An Lei, Zhi-Hao Huang, Yun Jin, Lei Li, Ning Su, Ya-Lin Xie, Zhen-Gang Zhao, Jian-Xiong Liu

**Affiliations:** ^1^Department of Oncology, Guangzhou Chest Hospital, Guangzhou, China; ^2^Department of Pathology, Guangzhou Chest Hospital, Guangzhou, China; ^3^Department of Tuberculosis Oncology, Guangzhou Chest Hospital, Guangzhou, China; ^4^School of Food Science and Engineering, South China University of Technology, Guangzhou, China; ^5^Department of Thoracic Surgery, Guangzhou Chest Hospital, Guangzhou, China

## Abstract

**Purpose:**

Studies on genetic alterations of the heterogenous small cell lung cancer (SCLC) are rare. We carried out the present study to clarify the genomic alterations and TMB levels of Chinese SCLC patients by whole-exome sequencing.

**Materials and Methods:**

Whole-exome sequencing by next-generation sequencing technique was implemented on twenty SCLC samples. Significant somatic mutations and copy number variations were screened, followed by comparison with the data extracted from COSMIC. Besides, altered signaling pathways were examined in order to figure out actionable targets.

**Results:**

A total of 8,062 nonsynonymous mutations were defined. The number of mutations for each case ranged from 98 to 864. As for base substitutions, a total of 15,817 substitutions were detected with C > A conversion which was correlated to smoking occupying 25.57%. The TMB values ranged from 2.51/Mb to 22.1/Mb with a median value of 9.95/Mb. *RB1* was the most frequently mutated gene altered in 18 (90%) cases, followed by *TP53* altered in 17 (85%) cases. Other commonly changed genes were *PTEN*, and *RBL1*, with frequencies of 55% and 50%, respectively. *SOX2* significantly amplified in 6 (30%) cases and *MYCN* amplified in 1 (5%) patient. Notch signaling pathway and PI3K/AKT/mTOR signaling pathway were universally and significantly changed. Major genomic alterations were in consistency with data from COSMIC, but frequencies of less common mutations were different.

**Conclusion:**

*TP53* and *RB1* inactivations were universally detected in SCLC. The Notch and PI3K/AKT/mTOR signaling pathways were both significantly altered, implying potential actionable targets.

## 1. Introduction

In 2012, there were an estimated 1,800,000 new lung cancer cases all over the world [1]. And lung cancer remains the leading cause of death in both male and female from more developed countries. The major subtype of lung cancer is non-small cell lung cancer (NSCLC), accounting for around 85% of all lung cancer incidences [2]. The genomic landscape of NSCLC has been studied comprehensively, based on which various kinds of targeted agents were being developed [2–4].

Unlike NSCLC, small cell lung cancer (SCLC), which accounts for approximately 10–15% of all lung cancers, is not understood as well, and targeted therapy for SCLC is lacking. SCLC is a poorly differentiated neuroendocrine carcinoma, which is highly aggressive with about 75% patients presented with metastases at the time of diagnosis or diagnosed at extensive stage [5]. Therefore, SCLC is rarely treated with surgery, and few tissue specimens are available for genomic variation detection. A majority of SCLCs are susceptible to chemotherapy and radiotherapy, but disease recurrence or progression can take place soon after leaving the patients killed in months [6]. To develop effective targeted therapies for SCLC, it is critical to illustrate the genomic variations of this cancer.

A few studies based on Caucasian population identified universal aberrations of *TP53* and *Rb1* in SCLC as well as some actionable genomic variations like *KRAS* and *BRAF* mutations [7–9]. Genome-wide screening data from Asians were much less. To our knowledge, there was only one study from Japan that characterized the genomic changes of 51 SCLC samples by whole-exome sequencing [10]. No significant differences were found when the universal inactivation of *TP53* and *Rb1* was considered, which was regarded as hallmarks of SCLC. However, other less frequently mutated genes varied from one study to another. Hence, there is still a huge gap needing to be filled before a comprehensive genomic variation of SCLC is clarified. It is necessary to delineate the genomic alterations of more SCLC specimens from different populations especially those of Asian and Black.

Therapeutic options for SCLC remained unchanged for almost three decades. Recently, the application of immune checkpoint inhibitors (ICI) provides SCLC patients with a new choice, especially when those agents were applied in combination [11]. In consistency with other tumors, the efficacy of ICI was low in the unchosen population, whereas, when SCLC patients were stratified according to tumor mutation burden (TMB), a significantly prolonged progression-free survival (PFS) and overall survival (OS) were achieved in ICI combination group [11]. SCLC has a relatively high TMB level which may partly explain the treatment effect of ICI [12]. Genomic alterations that are associated with different TMB levels are not defined in SCLC. Besides, reproducible molecular biomarkers for SCLC prognosis are still lacking [13].

We carried out the present study to clarify the genomic alterations and TMB levels of Chinese SCLC patients by whole-exome sequencing. We also attempted to better understand the correlations of genomic alterations with TMB levels, clinical outcomes in SCLC, and identify candidate prognostic biomarkers. Moreover, we tried to figure out whether or not there were significant differences between our results and the mutational data from Catalogue of Somatic Mutations in Cancer (COSMIC) database.

## 2. Materials and Methods

### 2.1. Patients and Samples

From January 2017 to December 2018, a total of twenty SCLC tumor and matched normal lung Formalin-Fixed and Paraffin-Embedded (FFPE) tissue samples were obtained from Guangzhou Chest Hospital. Clinicopathological information were retrospectively collected. The stages of all SCLC participants were classified into limited-stage and extensive stage according to the older Veterans Administration Lung Study Group's 2-stage classification scheme [14]. Informed consent was obtained from all patients or their authorized relatives. This study was approved by Ethics Committee of Guangzhou Chest Hospital and carried out in compliance with the Declaration of Helsinki Principles.

### 2.2. DNA Extraction

We performed DNA extraction from serial thick sections cut from tumor tissue samples and control sections. The invasive tumor content was estimated by pathologists to ensure more than 50% of the cells were tumor cells. DNA was isolated from the FFPE tissue samples using the DNeasy Blood and Tissue Kit (69504, QIAGEN, Venlo, Netherlands).

### 2.3. Next-Generation Sequencing

We performed whole-genome sequencing using the technique of next-generation sequencing in order to detect the genomic alterations of those tissue specimens. First, targeted capture pulldown and exome-wide libraries from native DNA were created using the TruePrep DNA Library Prep Kit V2 for Illumina (#TD501, Vazyme, Nanjing, China). Then, generated paired-end sequence data using Illumina HiSeq machines. Exome Research Panel-Integrated DNA (Tongshu BioTech, Shanghai, China) was applied to accomplish WES. Average sequencing depth of coverage was greater than 250x, and more than 99% exomes had sequencing depth of >100x.

### 2.4. Data Analysis

The sequencing data were firstly aligned to the human reference genome (NCBI build 37) using BWA, and then sorted followed by PCR duplication removal using GATK 4.0. Firstly, paired normal tissues were sequenced and data were used to screen out germline mutations. Then, somatic mutation calling was performed using Mutect1, Strelka2, and VarDict. Somatic mutations fell in at least two of the results of the three software were selected as high confidential mutations. As for the inactive mutations in TP53 and RB1, the provided manual list of variant classifications are considered as nonsynonymous. The rest will be considered as silent variants. Default uses Variant Classifications with High/Moderate variant consequences (http://asia.ensembl.org/Help/Glossary?id=535:“Frame_Shift_Del,”“Frame_Shift_Ins,”“Splice_Site,”“Translation_Start_Site,”“Nonsense_Mutation,”“Nonstop_Mutation,”“In_Frame_Del,”“In_Frame_Ins,”“Missense_Mutation.”). Copy number variations (CNV) and loss of heterozygosity (LOH) were analyzed using CNVkit. The somatic mutations in each patient's samples were annotated with ANNOVAR, and the annotated files were converted to an MAF file using maftools. The oncoprint was drawn using nonsynonymous mutations with maftools. Pairwise comparisons of CNV and LOH were performed using ggpubr package. TMB values were calculated as dividing the number of nonsynonymous mutations by the size of sequencing panel. Significant mutations were furtherly selected as described previously [4].

VCF files of mutational data and basic information were downloaded from COSMIC. Patients diagnosed as SCLC were selected and respective mutational data were extracted. Selected mutational data were normalized by bcftools before data analysis was carried out. Data analysis was performed in the same way as our own sequencing data. Mutated genes and their frequencies from COSMIC were calculated and compared with that of our cases.

### 2.5. Statistical Analysis

Measurement data were analyzed by Mann–Whitney *U* test, and categorical data were compared using Fisher exact probability test. Survival data were calculated using the Kaplan–Meier method. All tests were bilateral, with *p* < 0.05 indicating difference with statistical significance. Statistical analysis was carried out by the statistical software package SPSS 22.0 (IBM Corp., Somers, NY, USA).

## 3. Results

### 3.1. Clinicopathological Characteristics of SCLC Patients

The present study enrolled a total of twenty patients, among whom 19 were males and only one patient was female. All of them were Asians. The ages of the patients ranged from 24 to 78 years with a median age of 64 years. 15 cases presented a smoking history. Additionally, 18 patients received etoposide plus cis-platinum doublet chemotherapy, 1 had paclitaxel monotherapy, and 1 did not receive any chemotherapy. More details of basic information are revealed in Table 1.

### 3.2. An Overview of the Genomic Alterations of 20 SCLC Samples

**Figure 1:**
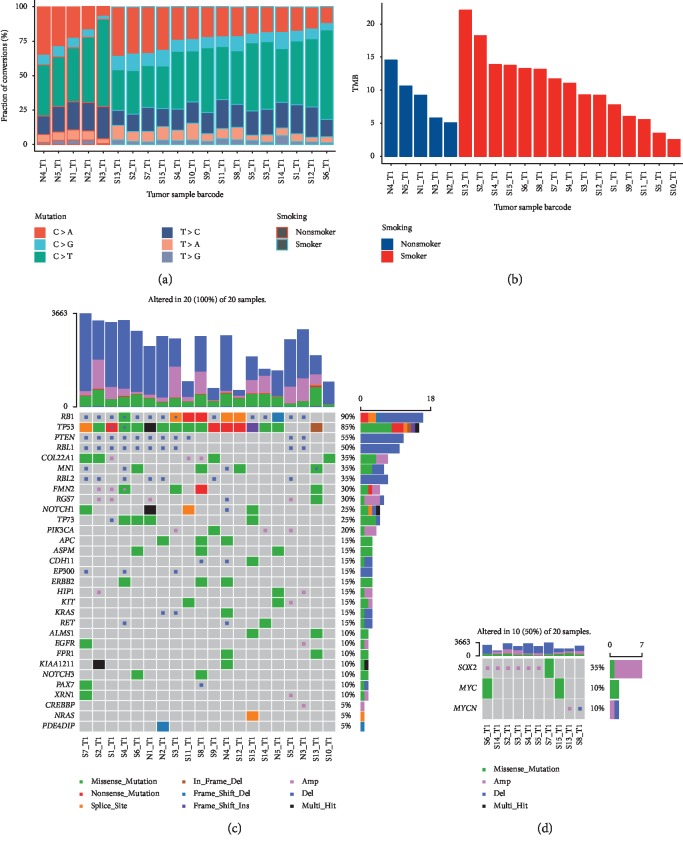
Genomic alterations in the 20 patients of the current study. (a) The base substitution fractions. Five cases on the left side are nonsmokers, the other fifteen patients are smokers. (b) The TMB values of those patients, where blue bars indicate nonsmokers and red bars indicate smokers. (c) An overview of significantly mutated genes. Genes were listed top down according to aberration frequencies. (d) Significantly amplified genes.

Genomic alterations in the 20 patients of the current study are shown in Figure 1. A total of 8,062 nonsynonymous mutations were defined, which included 7,289 missense mutations, 422 nonsense mutations, 14 nonstop mutations, 125 splice sites, and 212 indels. The number of mutations for each case ranged from 98 to 864 with a majority of missense mutations (range 90–742 per case). As for base substitutions, a total of 15,817 substitutions were detected, among which 4,035 were C > A substitution. C > A conversion which was correlated to smoking occupied 25.57%, whereas C > T associated with nonsmoking occupied 39.82%. The median value of C > A substitution in nonsmoking people (*n* = 5) was 155 (range 24–377 per sample), while that in smoking people (*n* = 15) was 130 (range 43–637 per sample). No statistically significant difference was found (*p*=0.727). The median value of C > T substitution in nonsmoking people was 276 (range 159–408 per sample), while that in smoking people was 340 (range 67–582 per sample). Still, no statistically significant difference was found (*p*=0.407). Detailed base substitution fractions are shown in Figure 1(a). Five cases on the left side are nonsmokers; the other fifteen patients are smokers.

The TMB values ranged from 2.51/Mb to 22.1/Mb with a median value of 9.95/Mb. The median TMB value of nonsmokers was 9.23/Mb, and that of smokers was 11.05/Mb. No statistically significant difference was found between smokers and nonsmokers (*p*=0.541). The column graph Figure 1(b) displays the TMB values of those patients, where blue bars indicate nonsmokers and red bars indicate smokers.

An overview of significantly mutated genes is depicted in Figure 1(c). Genes were listed top down according to aberration frequencies. *RB1* was the most frequently mutated gene altered in 18 (90%) cases, followed by *TP53* altered in 17 (85%) cases. Other genes changed in more than half of those cases were *PTEN*, and *RBL1*, with frequencies of 55% and 50%, respectively.  Figure 1(d) shows significantly amplified genes. *SOX2* significantly amplified in 6 (30%) cases and *MYCN* amplified in 1 (5%) patient.

**Figure 2:**
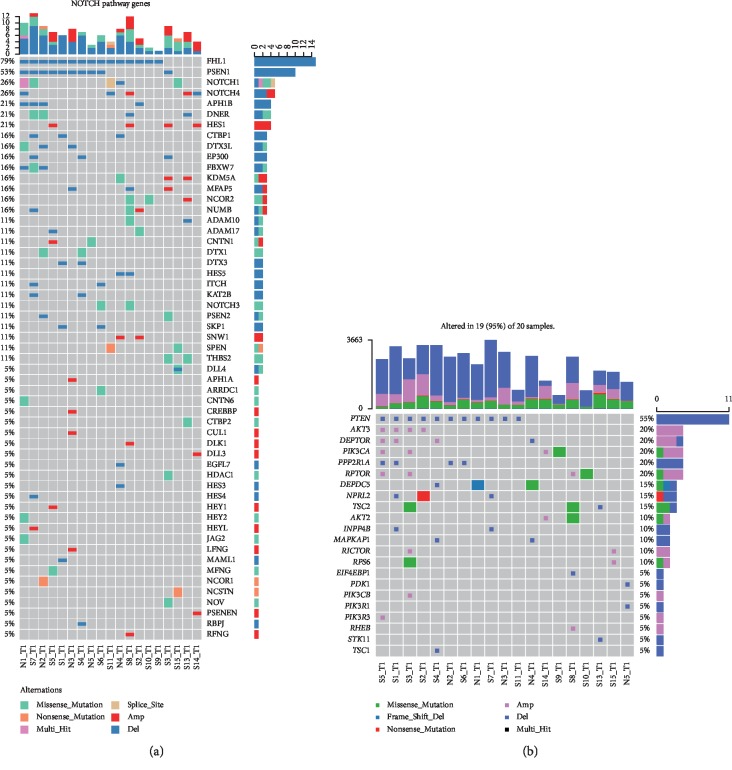
Important signaling pathways screened out in our cases. (a) The genetic changes of Notch signaling pathway and (b) that of PI3K/AKT/mTOR signaling pathway.

We screened out two pathways, Notch signaling pathway and PI3K/AKT/mTOR signaling pathway, of which the gene members were universally and significantly changed. Important signaling pathways screened out in our cases are depicted in Figure 2.  Figure 2(a) displays the genetic changes of Notch signaling pathway and Figure 2(b) shows that of PI3K/AKT/mTOR signaling pathway.

### 3.3. Comparison with COSMIC

**Figure 3:**
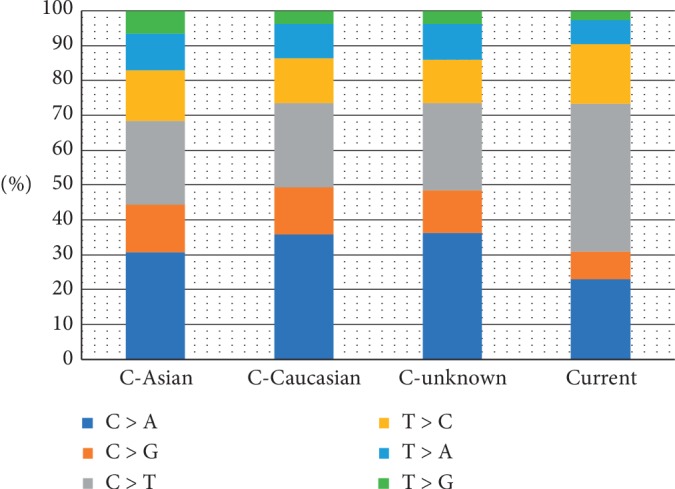
Comparison of base substitution fractions between our patients with cases from COSMIC. Cases from COSMIC were furtherly stratified by race. C is for COSMIC and current represent the present study.

To compare the genetic alterations of our patients with data from COSMIC, we downloaded and analyzed the whole-exome screening data in COSMIC. As a result, a total of 265 cases were extracted, which included 32 Asians, 90 Caucasians, 1 Black, and 142 unknown Ethnics. The number of mutations ranged from 1 to 942 per sample of SCLC cases in COSMIC examined on a whole-exome scale. The frequencies of C > A substitution were similar among different races in COSMIC, the same with C > T substitution. However, the frequency of C > A substitution in our samples was much lower, while the fraction of C > T substitution was higher than that in COSMIC. Comparison of base substitution fractions between our patients with cases from COSMIC is presented in Figure 3. Cases from COSMIC were furtherly stratified by race. C is for COSMIC and current represent the present study.

The TMB values of cases in COSMIC ranged from 0.03/Mb to 19.31/Mb with a median value of 2.58/Mb. No statistically significant differences were found between different races (*p*=0.112). Nevertheless, the TMB values of cases in COSMIC were statistically lower than that of the 20 cases from our healthcare center (*p* < 0.001).

The frequencies of significant genetic aberrations were different between our cases and cases in COSMIC. Mutational information of cases in COSMIC was presented in Supplementary [Supplementary-material supplementary-material-1], compared with that of our cases in Figure 1(c). As was shown, *NRAS* splice aberration was exclusively detected in one case of our study, whereas *ROS1* and *PTGFRN* alterations were exclusively screened out in cases from COSMIC. No pathogenetic amplifications of *SOX2*, *MYC*, and *MYCN* were found in the cases from COSMIC.

The fractions of base conversions and TMB values of cases in COSMIC are detailed in Supplementary Figures [Supplementary-material supplementary-material-1] and [Supplementary-material supplementary-material-1], respectively.

### 3.4. Progression-Free Survival (PFS) and Overall Survival (OS)

**Figure 4:**
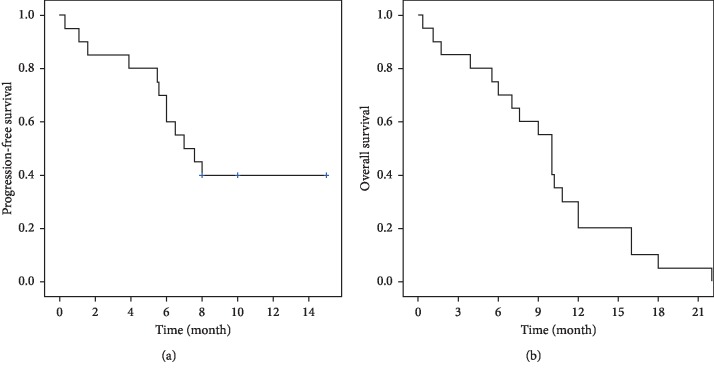
The Kaplan–Meier curves of patients' progression-free survival and overall survival.

As shown in Table 1, all but two SCLC patients received first-line standard platinum-based doublet chemotherapy. Of these two exceptional cases, one received paclitaxel monotherapy, while the other did not receive any therapy at all. The median PFS of the 20 cases was 7.3 months (range 0.3–15 months), and the median OS was 9.5 months (range 0.3–22 months). Survival plot is depicted in Figure 4.  Figure 4(a) shows Kaplan–Meier curve of progression-free survival.  Figure 4(b) displays Kaplan–Meier curve of overall survival.

## 4. Discussion

SCLC differs from NSCLC in clinical manifestations, pathological features, and genomic landscape. In contrast to the deep understanding of oncogenesis and highly progressed treatment agents of NSCLC, no significant progress has been achieved in SCLC during the past three decades [15]. The main reason of this discrepancy is the lack of tissue samples for examination. Several studies were carried out focusing on the genomic alterations of SCLC [7–9]. However, considering the number of SCLC cases in clinical settings, studies were rather sparse and small in size, especially in Asian population [10]. Another study detecting the molecular profiles of SCLC was based on targeted mutation sequencing rather than whole-genome or -exome screening [16]. Our study delineated the genomic aberrations and TMB values of twenty SCLC Asian patients with different clinical features by performing whole-exome sequencing. Besides, we compared the genomic alterations and TMB values between SCLC patients in different groups stratified by their clinical features. Furthermore, we compared our findings with data in COSMIC. We found that the major genomic alterations in our cases were in consistency with previous studies, but frequencies of less common mutations were different. Our results served as a supplement to the genomic landscape of SCLC. We also found crucial signaling pathway alterations that may offer actionable targets.

SCLC is a type of genetically instable cancer. The major subtype of its base substitutions was C > A, accounting for over 30% of all substitutions, which indicated the role of smoking exposure to SCLC pathogenesis [15]. The fraction of C > A conversion in our cases was lower than cases from other studies and COSMIC. Within COSMIC, Average C > A percentage of Asian patients was lower than Caucasian patients. These findings indicate diverse genetic reactions of different populations to smoking. Previous studies demonstrated a universal inactivation of *TP53* and *RB1* in SCLC, which were regarded as the initiating and crucial events in SCLC oncogenesis [7–10]. Similar to those researches, the inactivation of *TP53* and *RB1* occurred in 85% and 90% of our cases, respectively. Besides, many other genes also repeatedly altered in SCLC, such as *EP300*, *PTEN*, *ERBB2*, and *CREBBP* [7–10]. Most of these previously known mutations were detected in our study, but the frequencies were different, indicating a heterogenous genomic landscape of SCLC among different races or ethnics. This phenomenon was also seen when we compared our results with the data from COSMIC. For now, COSMIC was the only available database for SCLC mutational landscape [17]. However, given the heterogeneity feature of SCLC, the number of enrolled cases was considerably small. It would be better if more SCLC mutational data were added. The TMB levels of the patients in our study were statistically higher than that of the cases in COSMIC database, which may be caused by a sampling bias, while no statistically significant difference in TMB values was found between smokers and nonsmokers in our study, probably due to the small sample size of this study.

The Notch and the PI3K/AKT/mTOR signaling pathways were both significantly altered in our study. The Notch signaling pathway has long been proved to be a key player in multiple cellular processes, such as proliferation, differentiation, and stem cell maintenance [18]. Unlike in NSCLC where Notch signaling pathway acts as an oncogene, it acts as a suppressor gene in SCLC. Inactivation of Notch pathway was found in a majority of SCLCs. And the role of Notch pathway in SCLC was highly complex [15]. Agents targeting key factors of this pathway are being developed [19]. With the clarification of Notch in SCLC, more therapeutic targets may be offered. Another signaling pathway that may provide potential therapeutic targets is the PI3K/AKT/mTOR pathway. PI3K/AKT/mTOR signaling pathway is validated to be involved in the migration, proliferation, and survival of SCLC cell lines [20]. Changes of molecules in this pathway were also detected in about 40% of clinical SCLC samples [10]. In our study, 95% of the 20 SCLC specimens possess abnormal PI3K/AKT/mTOR signaling pathway. Various targeted drugs have been developed or are on the way of being developed aiming to block PI3K/AKT/mTOR signaling pathway, which suggested a possibility of target therapy for SCLC.

Even though molecular factors associated with SCLC prognosis were not able to be defined due to the small sample size of the current study, one special case attracted our attention. He was 55 years old with a smoking history of 20 pack-year when he was initially diagnosed as SCLC staged at T3N3M0. Among all 20 participants in our study, he had the longest PFS (15 months) and OS (22 months). WES results showed wildtype *TP53* and *RB1* accompanied by a TMB value of 2.51/Mb, which was the lowest mutation load in our study. To find out the potential driver mutations in this case, we screened the mutations furtherly and got two candidate mutated genes, *WNT9A* and *ERAS*. *WNT9A* is a member of *WNT* gene family and correlated with oncogenesis [21]. *ERAS* may be involved in the pathogenesis and chemotherapy resistance of some types of cancers [22, 23]. Our study indicated their role in SCLC oncogenesis where *TP53* and *RB1* inactivation are absent. Besides, *TP53* and *RB1* wildtype may be correlated to a lower TMB level and act as a positive prognostic factor for SCLC. Large-scale researches are needed to validate the driver mutations in *TP53* and *RB1* wildtype SCLC and to figure out whether SCLC patients with wildtype *TP53* and *RB1* have better clinical outcome.

Our study supplements the genomic landscape of SCLC and suggests some potential actionable targets and prognostic factors. Nevertheless, there are three limitations within the current study. First, this is a small size retrospective study and there are biases considering the clinical features of the enrolled participants. So, these results cannot represent the genomic alterations of the whole Chinese SCLC population. Second, RNA sequencing was not performed because of limited sample mass, leaving the actual changes of signaling pathways unconfirmed. Third, we failed to figure out particular mutations associated with SCLC prognosis due to the small sample size.

## 5. Conclusion

In summary, *TP53* and *RB1* inactivations were universally detected in SCLC. The Notch and PI3K/AKT/mTOR signaling pathways were both significantly altered, implying potential actionable targets. Large cohort studies are needed to define comprehensive genomic aberrations of SCLC and validate potential actionable targets.

## Figures and Tables

**Table 1:** Baseline clinical features of SCLC patients.

Characteristics	All patients (*n* = 20) (%)
Age (median/range)	64/24–78 yr
≥60 yr	14 (70)
<60 yr	6 (30)

Gender	
Male	19 (95)
Female	1 (5)

Clinical stages	
Limited	3 (15)
Extensive	17 (85)

Smoking	
Non-smoker	5 (25)
Smoker	15 (75)

Histology	
SCLC	18 (90)
SCLC mixed with adenocarcinoma	2 (10)

Chemotherapy	
Etoposide and *cis*-platinum	18 (90)
Paclitaxel	1 (5)
None	1 (5)

## Data Availability

The clinical and genetic data used to support this article are included within the article and supplementary materials.

## Supplementary Materials

Supplementary Materials Supplementary Figure 1: the base substitution fractions of cases in COSMIC stratified by races. Supplementary Figure 2: the TMB values of cases in COSMIC stratified by races. Supplementary Figure 3: an overview of significantly mutated genes in cases from COSMIC.
