# Supplementary material for: Tumor Mutational Burden and Genomic Alterations in Chinese Small Cell Lung Cancer Measured by Whole-Exome Sequencing
Source: Biomed Res Int. 2019 Nov 6;2019:6096350. doi: 10.1155/2019/6096350 (PMC6874933; doi:10.1155/2019/6096350)
Supplement: Supplementary Materials — Supplementary Figure 1: the base substitution fractions of cases in COSMIC stratified by races. Supplementary Figure 2: the TMB values of cases in COSMIC stratified by races. Supplementary Figure 3: an overview of significantly mutated genes in cases from COSMIC. [file 6096350.f1.docx]

**
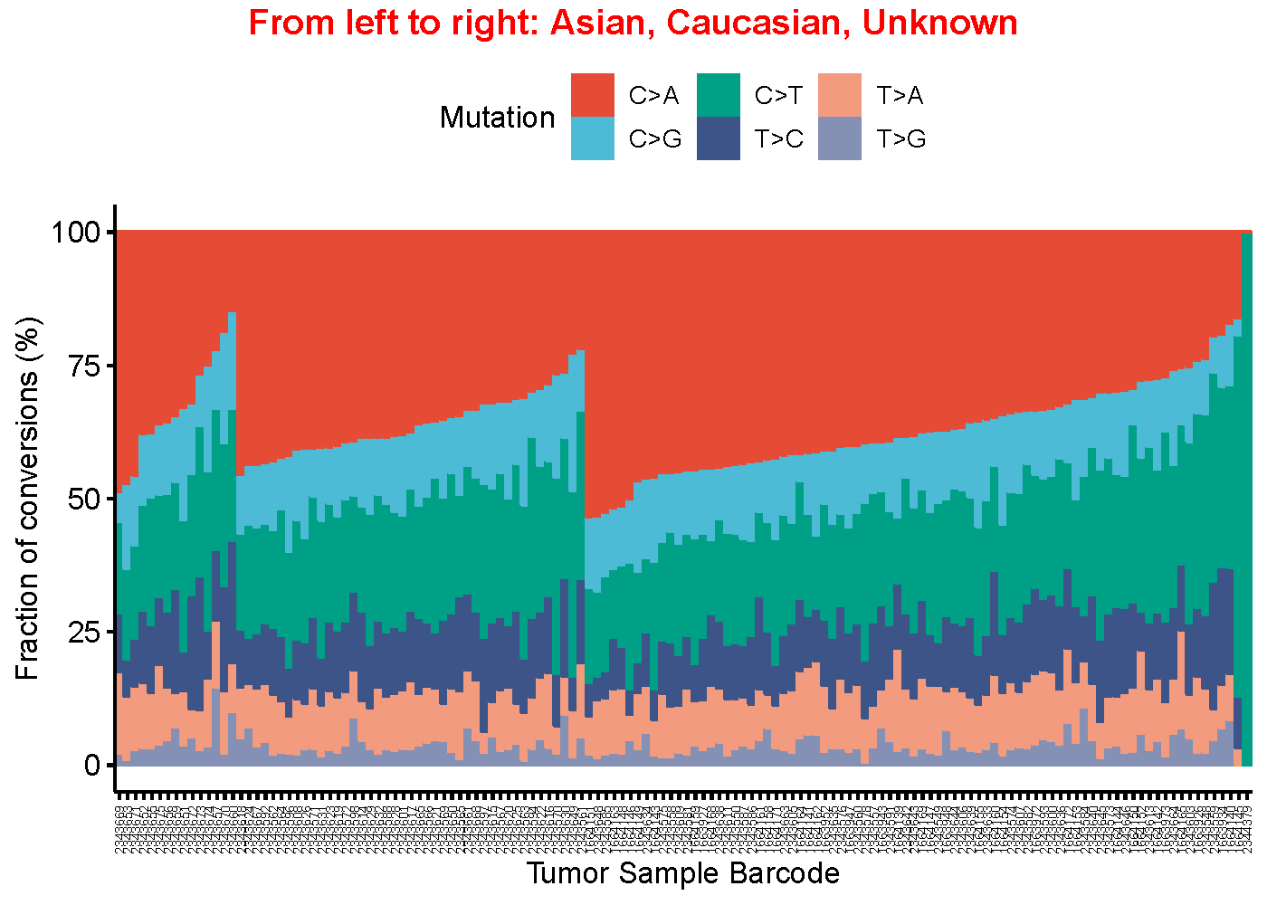
**

**Supplementary figure 1** The base substitution fractions of cases in COSMIC stratified by races.


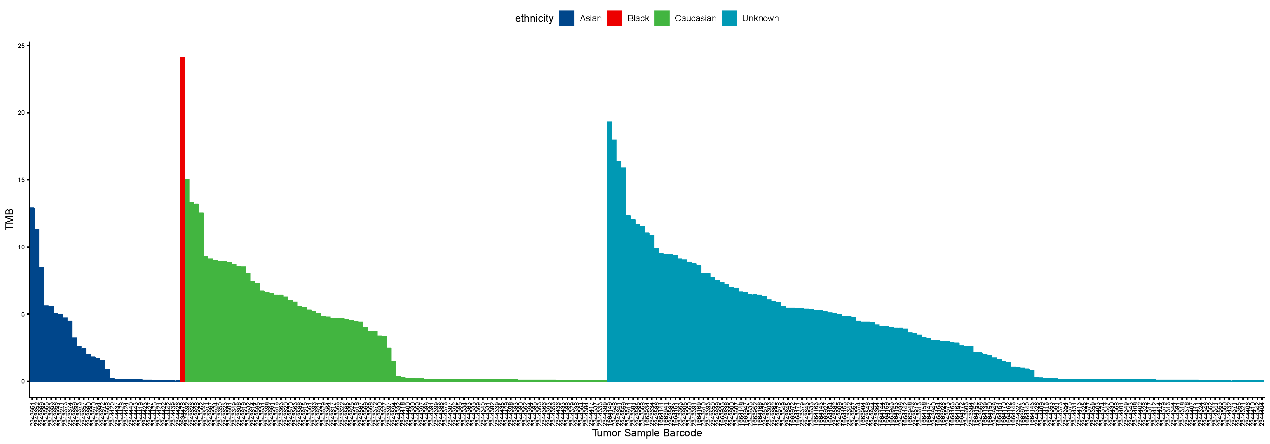


**Supplementary figure 2** The TMB values of cases in COSMIC stratified by races.


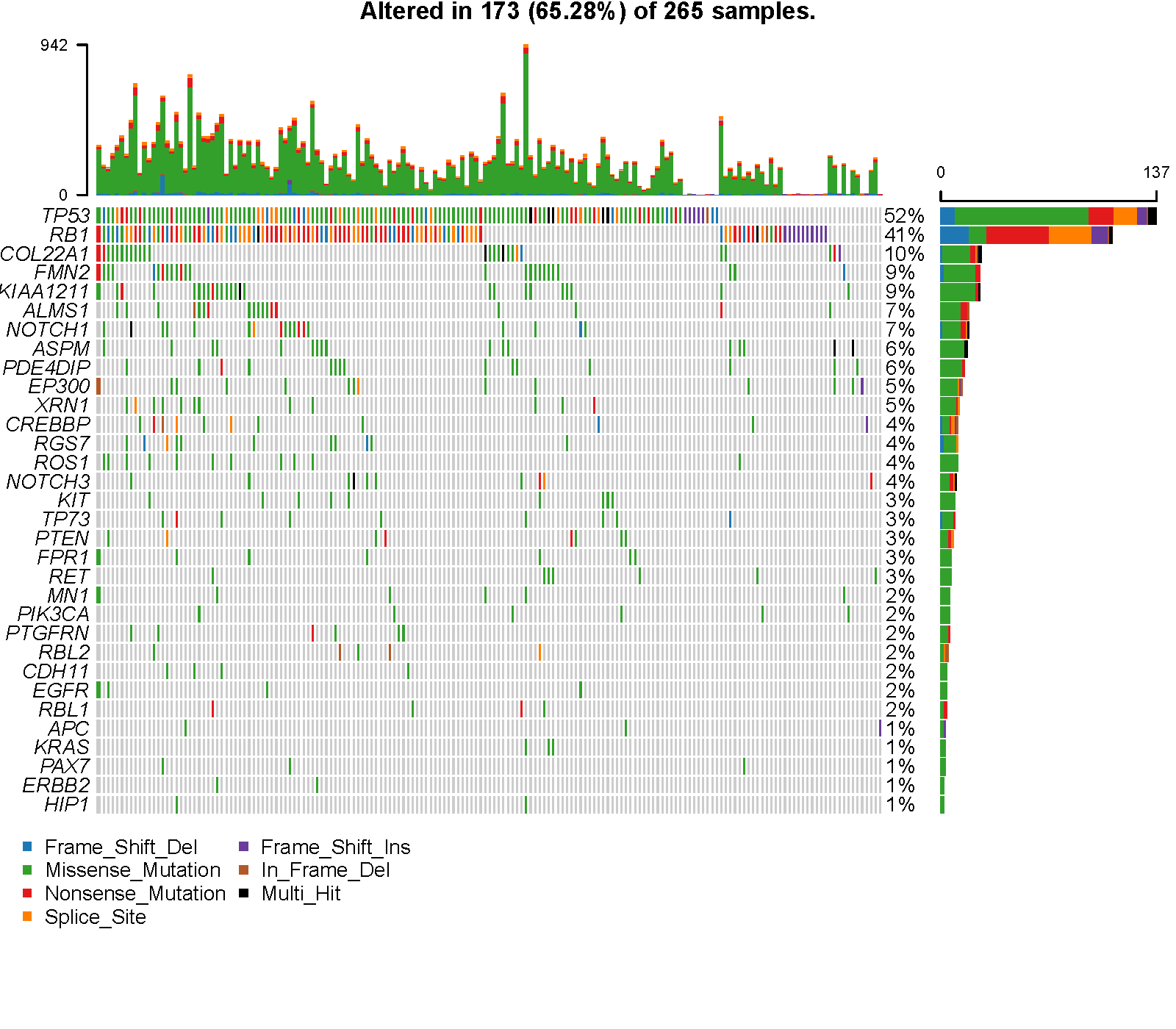


**Supplementary figure 3** An overview of significantly mutated genes in cases from COSMIC.
